# Suppression of adipocyte differentiation and lipid accumulation by stearidonic acid (SDA) in 3T3-L1 cells

**DOI:** 10.1186/s12944-017-0574-7

**Published:** 2017-09-25

**Authors:** Yueru Li, Yinghui Rong, Lisui Bao, Ben Nie, Guang Ren, Chen Zheng, Rajesh Amin, Robert D. Arnold, Ramesh B. Jeganathan, Kevin W. Huggins

**Affiliations:** 1 Department of Nutrition, Dietetics and Hospitality Management, Auburn University, Auburn, AL USA; 2 Department of Drug Discovery and Development, Harrison School of Pharmacy, Auburn University, Auburn, AL USA; 3 Boshell Diabetes and Metabolic Diseases Research Program, Auburn University, Auburn, AL USA

**Keywords:** Obesity, Stearidonic acid, Omega-3 fatty acids, 3T3-L1, Adipocyte differentiation

## Abstract

**Background:**

Increased consumption of omega-3 (ω-3) fatty acids found in cold-water fish and fish oil has been reported to protect against obesity. A potential mechanism may be through reduction in adipocyte differentiation. Stearidonic acid (SDA), a plant-based ω-3 fatty acid, has been targeted as a potential surrogate for fish-based fatty acids; however, its role in adipocyte differentiation is unknown. This study was designed to evaluate the effects of SDA on adipocyte differentiation in 3T3-L1 cells.

**Methods:**

3T3-L1 preadipocytes were differentiated in the presence of SDA or vehicle-control. Cell viability assay was conducted to determine potential toxicity of SDA. Lipid accumulation was measured by Oil Red O staining and triglyceride (TG) quantification in differentiated 3T3-L1 adipocytes. Adipocyte differentiation was evaluated by adipogenic transcription factors and lipid accumulation gene expression by quantitative real-time polymerase chain reaction (qRT-PCR). Fatty acid analysis was conducted by liquid chromatography-mass spectrometry/mass spectrometry (LC-MS/MS).

**Results:**

3T3-L1 cells treated with SDA were viable at concentrations used for all studies. SDA treatment reduced lipid accumulation in 3T3-L1 adipocytes. This anti-adipogenic effect by SDA was a result of down-regulation of mRNA levels of the adipogenic transcription factors CCAAT/enhancer-binding proteins alpha and beta (C/EBPα, C/EBPβ), peroxisome proliferator-activated receptor gamma (PPARγ), and sterol-regulatory element binding protein-1c (SREBP-1c). SDA treatment resulted in decreased expression of the lipid accumulation genes adipocyte fatty-acid binding protein (AP2), fatty acid synthase (FAS), stearoyl-CoA desaturase (SCD-1), lipoprotein lipase (LPL), glucose transporter 4 (GLUT4) and phosphoenolpyruvate carboxykinase (PEPCK). The transcriptional activity of PPARγ was found to be decreased with SDA treatment. SDA treatment led to significant EPA enrichment in 3T3-L1 adipocytes compared to vehicle-control.

**Conclusion:**

These results demonstrated that SDA can suppress adipocyte differentiation and lipid accumulation in 3T3-L1 cells through down-regulation of adipogenic transcription factors and genes associated with lipid accumulation. This study suggests the use of SDA as a dietary treatment for obesity.

**Electronic supplementary material:**

The online version of this article (10.1186/s12944-017-0574-7) contains supplementary material, which is available to authorized users.

## Background

Obesity is a complex disorder involving an excessive amount of body fat and has become one of the most serious health problems globally [1]. Although pharmaceutical interventions and surgical options have been adopted to address obesity, the usual primary strategy is through dietary modifications. Increased attention has been focused on dietary ω-3 polyunsaturated fatty acids (PUFAs), mainly EPA (20:5; ω-3) and DHA (22:6; ω-3), which have been reported to protect against the development of obesity and reduce body fat in humans [2, 3]. Cold water fish and fish oils are the most direct source of EPA and DHA to the body. However, due to concerns regarding fish palatability, sustainability, and food safety, there is a need to identify and develop alternative sources of EPA and DHA that have similar biological properties [4, 5].

**Fig. 1:**
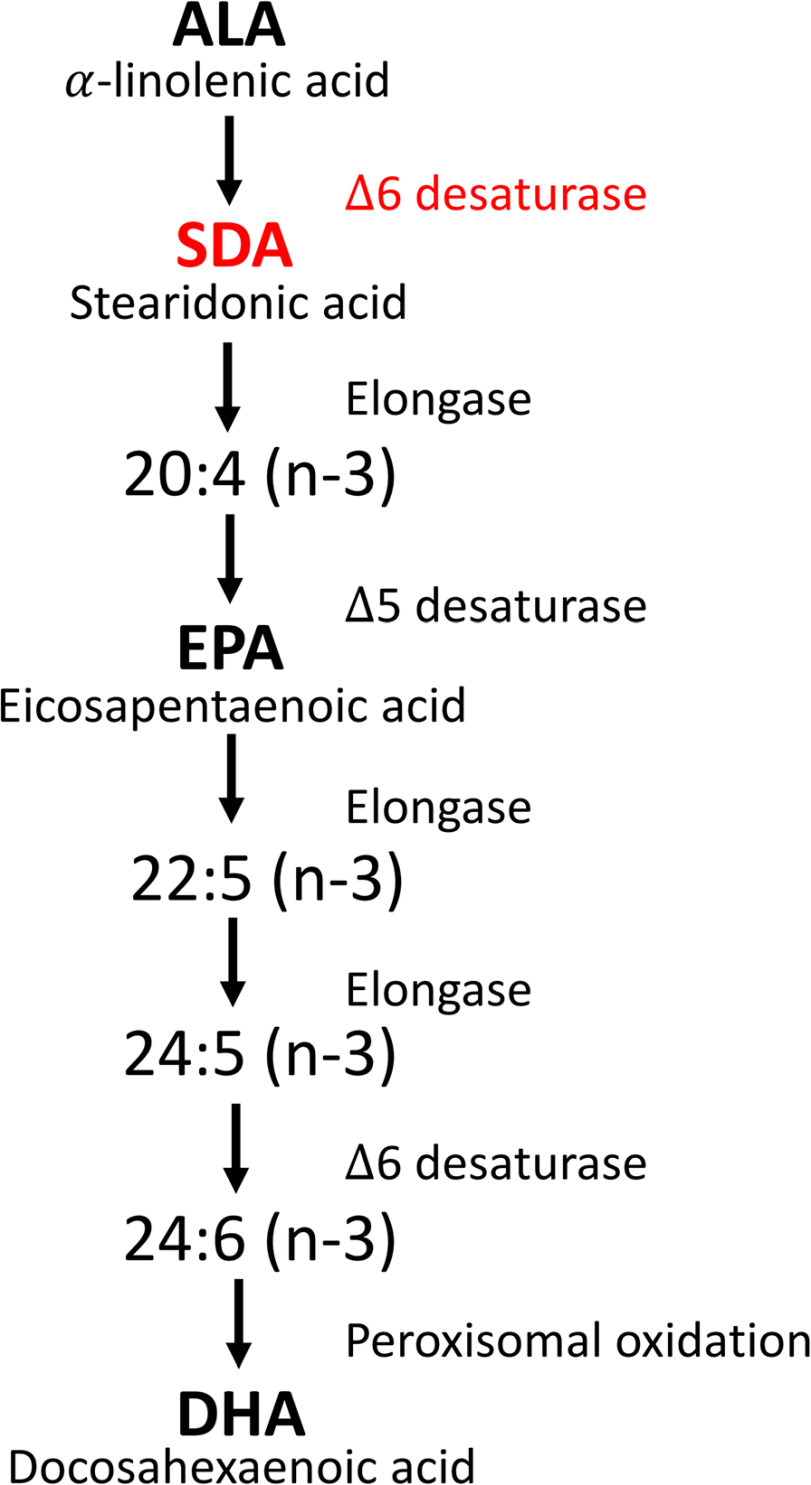
Metabolism of essential ω-3 fatty acids

Alpha-linolenic acid (ALA; 18:3; ω-3) is the main ω-3 fatty acid available in vegetable oils. Dietary ALA is capable of going through a series of desaturation and elongation reactions leading to its conversion to EPA and DHA (Fig. 1). However, due to limited activity of the rate-limiting enzyme-Δ6 desaturase for ALA substrate, the conversion to EPA and DHA is poor thus questioning the potential health benefits of ALA as a source of EPA and DHA [6]. Fortunately, consumption of stearidonic acid (SDA; 18:4; ω-3), the metabolic intermediate between ALA and EPA (Fig. 1), was found to result in significant EPA enrichment due to bypassing the ∆6 desaturase enzyme in ω-3 fatty acid metabolism [7]. Dietary SDA increased red blood cell EPA by approximately 17%, whereas the efficiency of ALA was about 0.1% [8].

SDA is found in the seeds and leaves of the *boragenase* plant family, such as echium, borage, evening primrose, and blackcurrant. Oils extracted from these plants are natural sources of SDA. Genetically modified SDA-enriched soybean oil with an improved SDA content is now available for research and commercial use [9]. Intake of SDA has been shown to significantly increase concentrations of long-chain ω-3 PUFAs in many tissues [10–12]. In addition, SDA has been observed to display similar biological functions to EPA and DHA. In the study by Kuhnt and colleges [12], healthy humans who consumed SDA (2 g/d) for 8 weeks, had improved lipid profile as evidenced by decreased serum levels of TG, cholesterol, low-density lipoprotein (LDL)-cholesterol, and oxidized LDL. Similar results were shown in a study with mild hypertriglyceridemia subjects, in which echium oil supplementation decreased plasma TG by an average of 21% compared to the baseline [13]. Additional studies have established the beneficial roles of SDA in dyslipidemia [14], inflammation [15], atherosclerosis [16], hepatic steatosis [10], cardiovascular disease [12], and cancer [17], suggesting SDA could be a new supplemental source of long-chain ω-3 PUFAs in health promotion and disease prevention.

Obesity is characterized at the cellular level by an increase in adipogenesis [18]. 3 T3-L1 cells have been used extensively as a cell culture model to study the molecular control of adipogenesis [19]. During 3 T3-L1 differentiation, a cascade of transcription factors is activated to modulate the expression of genes that are responsible for adipocyte development. Upon stimulation, C/EBPβ is first activated and directly induces the expression of C/EBPα and PPARγ, two key transcriptional regulators of adipocyte differentiation [20]. C/EBPα and PPARγ initiate a positive feedback loop to induce their own expression and playing pivotal roles by activating a large number of downstream target genes whose expression determines the phenotype of mature adipocytes [21]. These target adipogenic genes are mainly associated with cellular uptake of glucose and fatty acids, as well as TG hydrolysis and lipogenesis.

Long-chain ω-3 PUFAs, EPA and DHA, are known to inhibit adipocyte differentiation and decrease lipid accumulation by down-regulating the expression of certain transcriptional factors or lipolytic genes, such as C/EBP, PPARγ, SREBP-1c, AP2, FAS, SCD-1, and GLUT4 [22–26]. However, the effect of SDA on adipogenesis is unknown. Therefore, the present study hypothesizes that SDA will suppress adipocyte differentiation and reduce fat deposition in 3T3-L1 cells.

## Methods

### Cell culture

3T3-L1 mouse embryo fibroblasts were purchased from American Type Culture Collection (ATCC Manassas, VA) and cultured in humidified atmosphere of 5% CO_2_, 95% air at 37 °C. The cells were differentiated into adipocytes as previously described [27]. Briefly, 3 T3-L1 cells were maintained in a growth medium containing the following components: Dulbecco’s modified Eagle’s medium (DMEM) with high glucose, 10% fetal calf serum, and 1% penicillin-streptomycin. Two days after the cells reached confluence, differentiation was initiated by addition of differentiation medium (DMEM with high glucose, 10% fetal bovine serum, and 1% penicillin-streptomycin) along with the following components: 0.5 mM isobutylmethylxanthine, 1 μM dexamethasone, and 10 μg/mL bovine insulin (Sigma, MO). After another 3 days (Day 3), fresh differentiation medium containing only insulin was added for further 3 days until sample collection. All cell culture components were purchased from Invitrogen (Carlsbad, CA).

### Fatty acid treatment

Fatty acids (ALA, SDA, EPA, and DHA) were purchased from Matreya LLC, (State College, PA). Stock solutions of fatty acids were in ethanol and further diluted in DMEM containing 1.5% of fatty acid-free bovine serum albumin (BSA). After incubation at 37 °C for 1 h with constant shaking, fatty acid-supplemented medium and ethanol vehicle-control was applied to 3 T3-L1 adipocytes on Day 0. Cells were harvested on Day 3 and Day 6.

### Cell viability assay

3T3-L1 preadipocytes were treated with SDA (200 and 400 μM) or ethanol vehicle-control for 24 or 72 h from Day 0. Cell viability was determined by calcein-AM/propidium iodide staining with a commercial kit (Dojindo Molecular Technologies). Briefly, cells were suspended in 200 μl phosphate buffer saline (PBS) containing 1 mM calcein-AM and 1.5 mM propidium iodide. After incubation at 37 °C for 45 min, cells were pictured immediately under a Canon PowerShot S31S-attached Nikon TS100-F inverted microscope to assess the spatial distribution of the living cells in green (calcein staining) and dead cells in red (propidium iodide staining). In each well, at least three different random fields were examined. Live and dead cells in each field were quantified with NIS Elements Basic Research imaging software, and the percentage of cells with exclusively green fluorescence (interpreted as viable cells) was calculated.

### Oil red O staining

3T3-L1 cells differentiated for 6 days in the absence or presence of fatty acids were washed with PBS and then applied to Oil Red O staining assay according to the protocol of a commercial kit (Abcam, #133102). Briefly, cells were first washed with PBS and fixed with formalin solution for 15 min. The fixed lipid droplets were then stained with Oil Red O solution for 30 min at room temperature. Microscope images were taken to visualize red oil droplets staining in differentiated cells.

### Triglyceride accumulation assay

3T3-L1 cells differentiated for 6 days in the absence or presence of fatty acids were used to determine TG concentrations with a commercial kit (Abcam, #102513). Briefly, total cellular lipids were extracted with lipid extraction solution under heating. The TG content was then determined by adding lipase, which converted TG to glycerol. Glycerol was subsequently reacted to convert the probe to generate color, which can be measured spectrophotometrically at 570 nm in a plate reader. TG concentrations were calculated based upon a standard curve made from TG standards and normalized to total cellular protein content.

### Total RNA isolation and quantitative real-time PCR analysis

Total RNA was extracted using RNeasy Mini Kit (Qiagen; Valencia, CA) according to manufacturer’s instructions from 3T3-L1 cells differentiated for three or 6 days in the absence or presence of SDA. The quality and concentration of total RNA was determined spectrophotometrically using NanoDrop (Thermo Scientific). Complementary DNA (cDNA) was synthesized from 1 μg of RNA using iScript™ cDNA Synthesis Kit (Bio-Rad) according to the manufacturer’s protocol. Quantitative real-time PCR (qRT-PCR) was performed in the MyiQ single-color real-time PCR detection thermocycler (Bio-Rad) using iQTM SYBR® Green Supermix (Bio-Rad) to evaluate gene expression. Mouse gene specific primers were designed from Primer Bank and constructed by Integrated DNA Technologies, Inc. (IDT, Inc., Coralville, IA). Oligonucleotide sequences of the primers used for amplification are presented in Additional file 1: Table S1. The cycle threshold (ΔC_T_) method was used to measure relative quantification of the target gene, where values were normalized to the reference gene, 36B4. Fold changes of gene expression were calculated by the 2^-ΔΔCT^ method [28]. The statistical analysis was based on ΔC_T_ values.

### PPARγ transcriptional activity assay

The transcriptional activity of PPARγ was determined using the PPARγ ELISA kit (Cayman) according to the manufacturer’s protocol. Briefly, 3T3-L1 preadipocytes were treated with or without SDA for 24, 48, or 72 h, after which cells were washed with PBS and harvested with buffer A (20 mM HEPES, 10 mM KCl, 0.1 mM EDTA, 0.1 mM EGTA) containing protease inhibitor cocktail and PMSF (10 μL/mL). The cell lysate was centrifuged at 2500 × *g* for 5 min, and nuclear fractionate was then suspended in buffer B (0.4 M NaCl, 25% glycerol) containing protease inhibitor cocktail and PMSF (10 μL/mL). After incubation at 4 °C for 30 min, lysates were spun at 20,000 g for 30 min. The supernatant was then collected as nuclear protein fraction. The nuclear extracts were then incubated in wells coated with specific PPRE oligonucleotide sequences and further exposed to the primary anti- PPARγ antibody. Subsequently, the HRP-conjugated secondary antibody was added and the absorbance was quantified at 450 nm using a spectrophotometer. The result was normalized to total cellular protein content.

### Fatty acid analysis

3T3-L1 cells differentiated for 6 days in the presence of fatty acids or vehicle-control were used for fatty acid analysis. Lipid extracts from 3T3-L1 adipocytes were prepared using chloroform/methanol (C/M, 2/1, *v*/v). The organic phase was collected, dried under N_2_ gas, and dissolved in C/M (1/1, v/v). Saponification and formation of fatty acid methyl esters made from cellular lipids were then performed and measured by LC-MS/MS. Agilent 1290 UHPLC coupled Agilent 6460 QQQ triple quadrupole mass spectrometer was utilized to quantify the content of EPA and DHA within adipocytes. Palmitic acid-d31 (Sigma, purity >99%) was added as internal standard. Fatty acid content was normalized to total cellular protein content. Protein quantification was performed using the Bio-Rad DC Protein Assay Kit (Bio-Rad, Hercules, CA). BSA standard curve and sample preparation and analysis were realized according to manufacturer’s instructions.

### Statistical analysis

All data are presented as mean ± SD. The statistical significance of differences between groups was determined by one-way analysis of variance (One-way ANOVA) and Student’s t-test (two-tailed). The results were considered to be significant when the value of P was <0.05. Figures were produced by GraphPad Prism™ version 6.01 (GraphPad software, San Diego, CA).

## Results

### Effect of SDA on 3T3-L1 cell viability

**Fig. 2:**
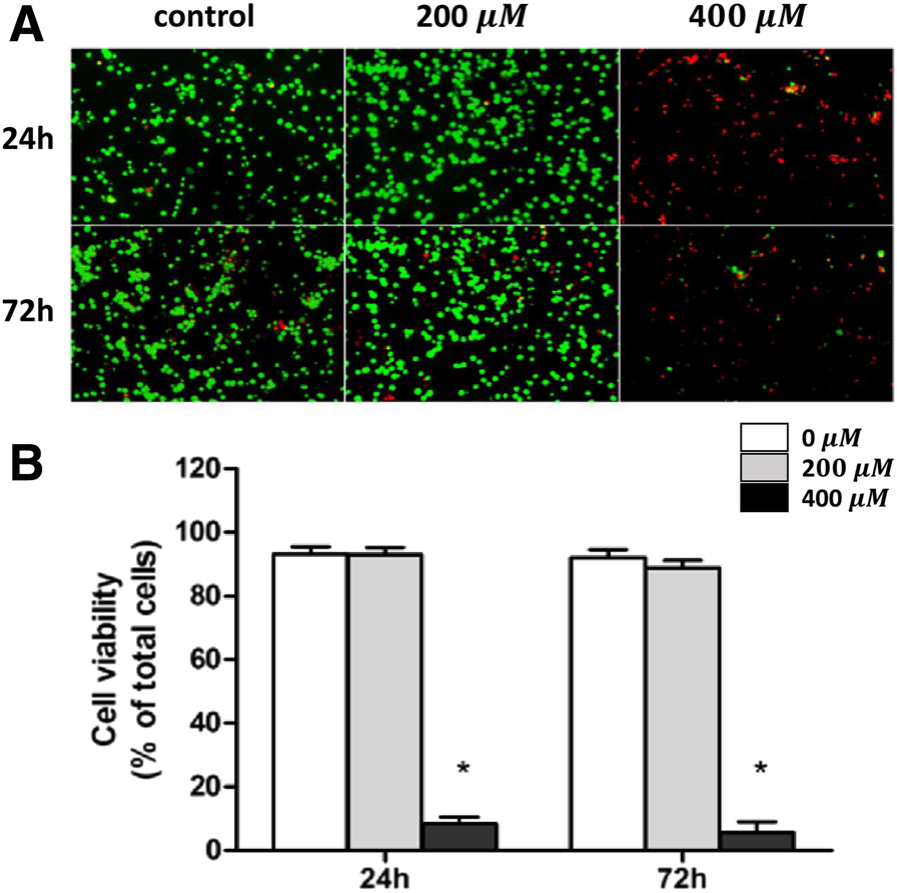
Effect of SDA on 3T3-L1 adipocyte viability. Two-day post-confluency preadipocytes were incubated with differentiation medium in the presence of SDA (0, 200, and 400 μM) for 24 or 72 h. Cell viability was detected by calcein-AM/propidium iodide staining. **a** A representative image of cell staining photographed using a microscope (X200). Living cells were stained with green (calcein staining) and dead cells were stained with red (propidium iodide staining). **b** Quantification of cell viability using spectrophotometry. Three images for each treatment were captured and analyzed. Values were obtained from three independent experiments and are expressed as the means ± SD; different from control cells: ^*^ *P* < 0.05

The concentration of 200 μM of ω-3 fatty acids were used in our experiments. Based on previous studies, 200 μM is a safe concentration for ALA, EPA, and DHA treatment in 3T3-L1 cells [29–31]. To assess that 200 μM of SDA was also safe for 3T3-L1 cells, cell viability assay was conducted. As shown in Fig. 2, the number of living cells (green) and dead cells (red) showed no statistically significant difference between controls and cells treated with 200 μM of SDA, no matter for 24 or 72 h. In contrast, 400 μM of SDA significantly decreased the cell viability to 8% (24 h) and to 5% (72 h) when compared to the control groups. These results indicate that the viability of 3T3-L1 cells was not affected by 200 μM of SDA. Therefore, the effect of SDA on adipocyte differentiation and lipid accumulation found in this study was independent of non-specific cell toxicity.

### SDA reduced lipid accumulation in 3T3-L1 adipocytes

**Fig. 3:**
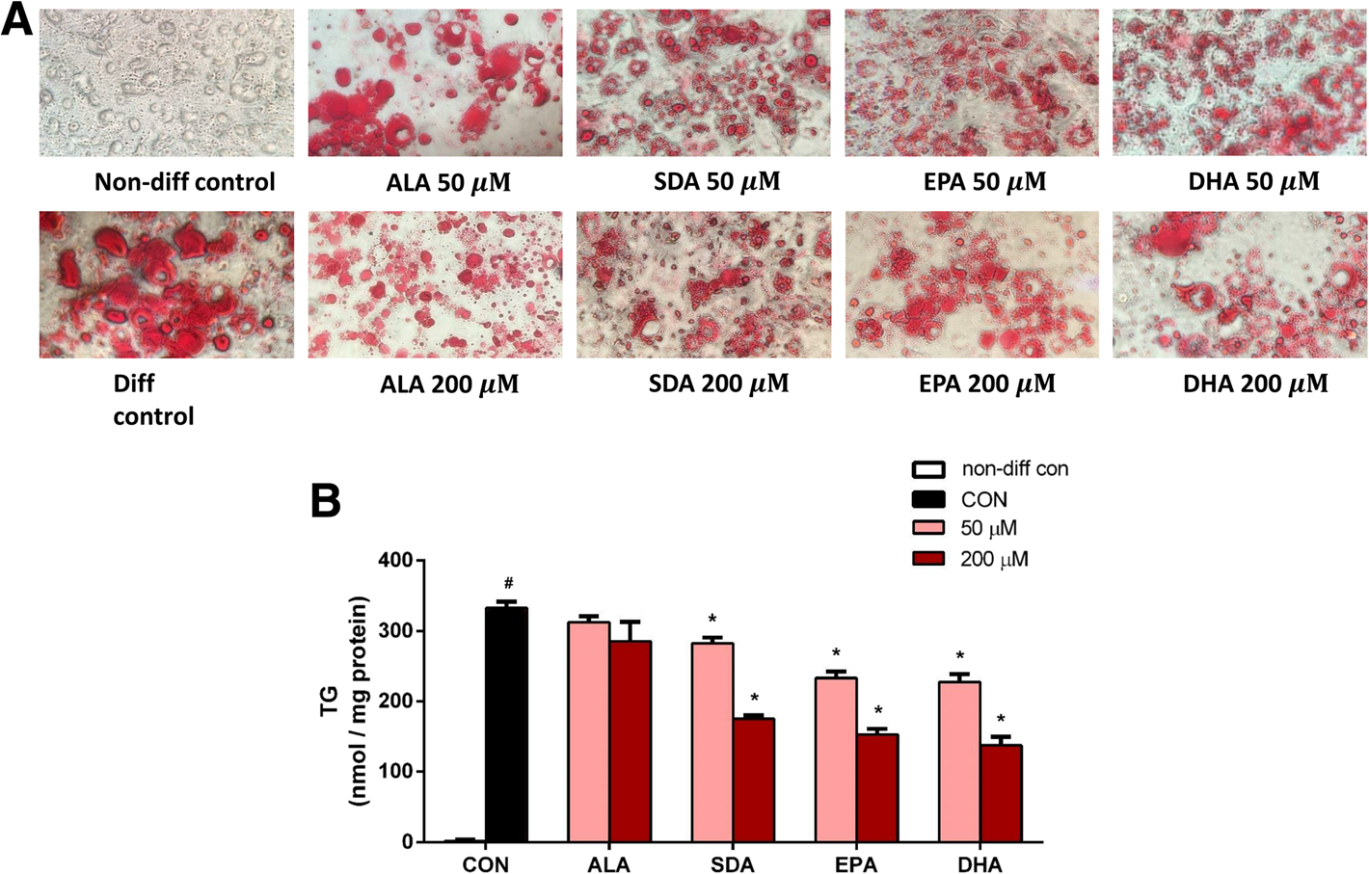
Effect of ω-3 fatty acids on TG accumulation in 3T3-L1 adipocytes. Two-day post-confluency preadipocytes were incubated with differentiation medium in the presence of ALA, SDA, EPA, and DHA (0, 50, and 200 μM) for 6 days. **a** Morphological observation and Oil Red O staining of 3T3-L1 cells photographed using a microscope (X200). Lipid droplets were stained in red. **b** Quantification of intracellular TG content. Data were obtained from three independent experiments. Absorbance value is given as mean ± SD; different from non-diff control cells: ^#^ *P* < 0.05; different from diff control cells: ^*^ *P* < 0.05

The effect of SDA and other ω-3 fatty acids on the storage of intracellular lipid in mature 3T3-L1 adipocytes was visualized by Oil Red O staining as shown in Fig. 3a. SDA, EPA, and DHA decreased the amount of lipid in 3T3-L1 adipocytes. This observed reduction in lipid accumulation was confirmed by TG quantification assay. As shown in Fig. 3b, compared to the differentiated control cells, 50 μM treatment of SDA, EPA, and DHA significantly decreased the TG content in 3T3-L1 adipocytes by 15, 30, and 31%, respectively. 200 μM treatment of SDA, EPA, and DHA significantly decreased the TG content by 47, 54, and 59%, respectively, compared to control cells. There was no significant effect on lipid accumulation in cells treated with ALA versus control cells.

### SDA down-regulates the expression of transcription factors and genes associated with adipogenesis and lipid accumulation

**Fig. 4:**
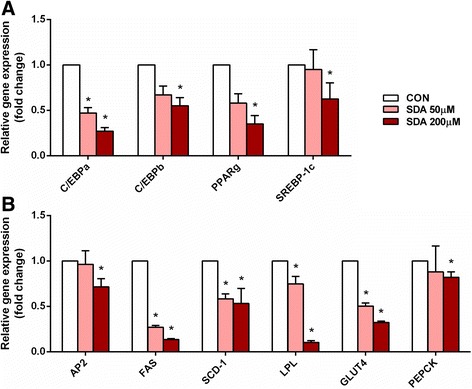
Effect of SDA on mRNA expression levels of adipogenic transcription factors and genes in 3T3-L1 adipocytes. Two-day post-confluency preadipocytes were incubated with differentiation medium in the presence of SDA (0, 50, and 200 μM). **a** The expression of C/EBPα, C/EBPβP, PPARγ, and SREBP-1c was evaluated by qRT-PCR with specific primer pairs on Day 3. **b** The expression of AP2, FAS, SCD-1, LPL, GLUT4, and PEPCK was evaluated by qRT-PCR on Day 6. The relative qRT-PCR values were corrected to 36B4 expression levels and normalized with respect to the control. Data were obtained from three independent experiments and are expressed as mean ± SD; different from control cells: *P < 0.05

To gain a better understanding of the molecular mechanism underlying the anti-adipogenic effect of SDA, the expression levels of key transcription factors involved in adipocyte differentiation and adipogenic genes involved in lipid uptake and metabolism were analyzed. The expression of transcription factors was measured on Day 3 after the stimulation of 3T3-L1 differentiation. As shown in Fig. 4a, SDA significantly decreased the mRNA expression of C/EBPα, C/EBPβ, PPARγ, and SREBP-1c in a dose-dependent manner. Compared to the vehicle-control cells, 50 μM SDA significantly decreased C/EBPα mRNA level by 50%. Increased SDA concentrations (200 μM) significantly decreased the mRNA expression of C/EBPα, C/EBPβ, PPARγ, and SREBP-1c by 73, 45, 65, and 37%, respectively, compared to the corresponding values in vehicle-control cells. Adipogenic gene expression was measured on Day 6 after the stimulation (Fig. 4b). At 50 μM SDA treatment, there was a significant reduction in FAS (73%), SCD-1 (42%), LPL (25%), and GLUT4 (50%) gene expression when compared to vehicle-control. At 200 μM SDA treatment, there was a significant reduction in AP2 (29%) FAS (87%), SCD-1 (47%), LPL (90%), GLUT4 (68%), and PEPCK (18%) gene expression when compared to vehicle-control cells.

### SDA inhibited the transcriptional activity of PPARγ during 3T3-L1 adipogenesis

**Fig. 5:**
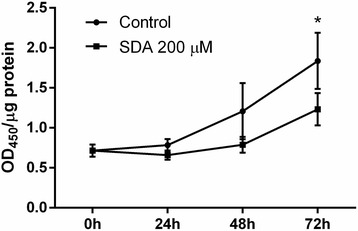
PPARγ binding activity in 3T3-L1 adipocytes treated with SDA. Two-day post-confluency preadipocytes were incubated with differentiation medium in the presence of SDA (0 and 200 μM) for 24, 48, and 72 h. ELISA-based PPARγ transcription factor assay was conducted to detect PPARγ binding activity to PPRE-containing double-stranded DNA sequences. Data were obtained from three independent experiments. Values are expressed as mean ± SD; different from control cells: *P < 0.05

Having demonstrated that SDA down-regulated the mRNA level of PPARγ during adipocyte differentiation, we determined whether SDA would affect the transcriptional activity of PPARγ. As shown in Fig. 5, SDA time-dependently inhibited PPARγ transcriptional activity in 3T3-L1 cells, and this inhibitory effect reached significant difference at Day 3. Consistent with the decrease in PPARγ gene expression, SDA significantly inhibited PPARγ transcriptional activity.

### SDA enriched EPA and DHA in 3T3-L1 adipocytes

**Fig. 6:**
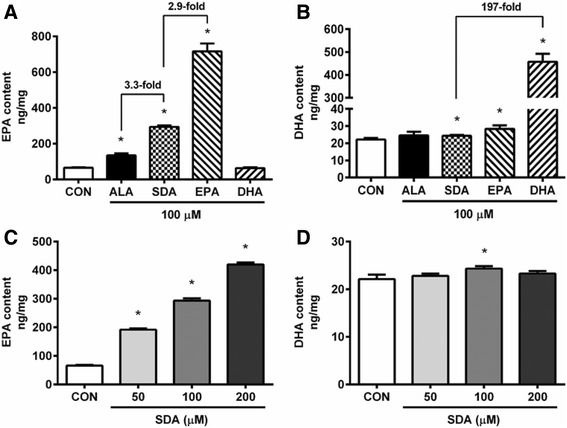
EPA and DHA enrichment in 3T3-L1 adipocytes treated with ω-3 fatty acids. Two-day post-confluency cells were incubated with differentiation medium in the presence of ω-3 fatty acids for 6 days. EPA and DHA content was measured by LC-MS/MS. **a** EPA enrichment by ethanol vehicle-control or 100 μM of ALA, SDA, EPA, and DHA in 3 T3-L1 cells. **b** DHA enrichment by ethanol vehicle-control or 100 μM of ALA, SDA, EPA, and DHA in 3T3-L1 cells. **c** EPA enrichment by SDA (0, 50, 100, and 200 μM) in 3T3-L1 cells. **d** DHA enrichment by SDA (0, 50, 100, and 200 μM) in 3T3-L1 cells. Values were obtained from three independent experiments and are expressed as the means ± SD; different from control cells: *P < 0.05

We investigated the metabolic fate of ω-3 fatty acids in 3T3-L1 cells differentiated in the presence or absence of ω-3 fatty acids. Previous studies have shown that there is efficient conversion of SDA to EPA and to a lesser extent DHA [32]. As shown in Fig. 6a, cellular content of EPA was significantly increased by 100 μM treatment of ALA, SDA, and EPA, by 103, 345, and 987%, respectively, compared to vehicle-control. The increased EPA content in SDA-treated cells was approximately one-third as effective as EPA itself. Compared to ALA, SDA treatment had a 3.5-fold more EPA enrichment in 3T3-L1 adipocytes. For DHA enrichment, 100 μM treatment of SDA, EPA, and DHA, significantly increased adipocyte DHA content by 10, 28, and 1967%, respectively, compared to vehicle-control (Fig. 6b). SDA increased adipocyte DHA was approximately 0.5% as effective as DHA. EPA treatment had minimal effect on the DHA level. ALA had no significant effect on DHA enrichment. To determine the dosage effect of SDA on EPA and DHA enrichment, 3T3-L1 cells were differentiated in the presence of 0–200 μM SDA. As shown in Fig. 6c, SDA increased EPA content in 3T3-L1 cells in a dose-dependent manner. DHA content was minimally affected by SDA treatment with a small but significant, increase at 100 μM SDA (Fig. 6d). This is consistent with other studies showing poor conversion of SDA to DHA [7]. Together, these results demonstrated that SDA was a better surrogate for EPA than ALA and indicated that SDA might be mediating its cellular effects through its conversion to EPA.

## Discussion

Long-chain ω-3 PUFAs, mainly EPA and DHA, have been found to reduce lipid accumulation in 3T3-L1 adipocytes through the regulation of adipogenic gene expression [22, 25]. SDA has been targeted as a potential biologically active surrogate for EPA as it can be effectively converted to EPA by the body and shares many beneficial properties with EPA [33]. In the present study, the effects of SDA on adipogenesis and lipid accumulation in 3T3-L1 cells were studied.

Adipogenesis and increased lipid accumulation are key features in obesity. Beneficial effects of EPA and DHA from fish oil against adipose tissue expansion and adipocyte differentiation were previously identified. Decreased TG content in 3T3-L1 adipocytes was found by treatment with high concentrations of sea cucumber and blue mussel extracts, which are rich in EPA and DHA [34], and also by treatment with EPA and DHA directly [35]. In line with these findings, we also observed a significant reduction in TG accumulation by EPA and DHA, as well as by SDA (Fig. 3). To our knowledge, this is the first study showing the influence of SDA on lipid accumulation in 3T3-L1 adipocytes. One study published opposite results, in which they found EPA and DHA promoted the differentiation of 3T3-L1 preadipocytes as evidenced by increased lipid content [36]. However, they treated the 3T3-L1 cells not only during differentiation, but also 24-h pretreatment before differentiation, so their results included both effects of EPA and DHA on differentiation and proliferation [36]. In our study, ω-3 fatty acids had an inhibitory effect on 3T3-L1 cell differentiation. It is not clear as to the effect of these ω-3 fatty acids on 3T3-L1 cell proliferation. In addition, our cells appeared to be more differentiated than theirs. As shown in Fig. 3, there is a large difference between non-differentiated control group and differentiated control group. However, in their study, a smaller number of 3T3-L1 preadipocytes were differentiated into mature adipocytes filled with lipid droplets based on their Bodipy staining and fluorescent images [36].

The differentiation of preadipocytes is regulated by a complex network of transcription factors, mainly the C/EBP family and PPARγ. SREBP-1c is implicated in stimulating endogenous PPARγ ligand production [37]. Adipocytes from mice in which the C/EBPα gene was disrupted showed defects in lipid accumulation [38]. Transgenic mice lacking PPARγ specifically in adipose tissue exhibited greatly reduced sized fat pads [39]. SDA has been shown to regulate gene expression [10, 33, 40, 41]. In the present study, it was hypothesized that SDA exerted anti-adipogenic effect by inhibiting the expression of these transcription factors and their target genes. As expected, dose-dependently down-regulation of C/EBPα, C/EBPβ, PPARγ, and SREBP-1c by SDA was observed (Fig. 4a). Consistent with the suppression of these adipogenic transcription factors, the expression of their target genes, AP2, FAS, SCD-1, LPL, GLUT4, and PEPCK were also significantly decreased dose-dependently by SDA treatment (Fig. 4b). The adipogenic genes are often connected with insulin sensitivity and inflammatory cytokines. AP2 has been demonstrated to act as an adipokine for the development of insulin resistance in liver [42]. Adipose-specific deletion of SCD-1 was found to induce GLUT1 up-regulation and was associated with decreased adiponectin and increased TNFα production [43]. Adipocyte-derived LPL was shown to induce macrophage activation and monocyte adhesion [44]. Inflammation has been shown to inhibit the expression of GLUT4 and PEPCK in 3T3-L1 adipocytes [45, 46]. EPA and DHA have been reported to have preventive properties in the development of insulin resistance and inflammation [47]. Therefore, it is necessary to further investigate the effect of SDA on adipokine secretion and its role in insulin resistance and inflammatory response for future study.

SDA is the Δ6 desaturase product of ALA in the bioconversion of ALA to EPA. In humans, the conversion of ALA to EPA is in low amounts (less than 7%) and in even lower amounts to DHA (less than 1%) due to the rate-limiting enzyme [48]. Nutritional supplementation with ALA was not able to induce the accumulation of long-chain ω-3 PUFAs. In the present study, it was hypothesized that by skipping the rate-limiting step, the conversion of SDA to EPA would be more efficient than that of ALA to EPA. By LC-MS/MS analysis, it was shown that SDA increased EPA content in 3T3-L1 cells by 345% compared to the control group (Fig. 6a). The efficacy of EPA enrichment by SDA was about 3.5-fold greater than the comparable level of ALA and was about one-third of the EPA enrichment by EPA. For DHA enrichment, both ALA and SDA, even EPA was not able to increase DHA content extensively in 3T3-L1 cells, although statistical significance was observed in SDA and EPA groups. This was probably due to another rate limiting step which converts docosapentaenoic acid to DHA [49]. Human studies have reported that consumption of SDA, as ethyl esters, echium oil, or SDA-soybean oil, significantly increased the EPA level in red blood cells [8], peripheral blood mononuclear cell [11], plasma [15], and neutrophils [13]. In animal studies, feeding with SDA was shown to increase the EPA content in many tissues, including red blood cells, plasma, liver, muscle, heart, brain, and ileum of dogs [50], sows and piglets [51], lambs [52], and rodents [53, 54]. In addition, SDA can lead to EPA enrichment in animal products, such as egg yolk, chicken meat [55], and milk of dairy cows [56]. Based on human studies, the efficacy of SDA on EPA enrichment in different tissues range from 17 to 85% as much as the efficacy of EPA treatment on EPA enrichment [8, 12, 57–60]. When compared to ALA, SDA was about 1.9 to 4.3-fold as effective as that of ALA on EPA enrichment [57, 61]. Most studies did not find significant change in DHA enrichment with SDA supplementation. These results indicate that SDA consumption will be expected to confer the health benefits associated with the consumption of EPA, but not DHA. While the exact molecular mechanisms by which SDA decreases gene transcription is not apparent from this study, the downstream biosynthesis of lipid-derived eicosanoids via its conversion to EPA may be a potential mechanism through which gene transcription effects could be modulated [25, 62, 63].

## Conclusion

**Fig. 7:**
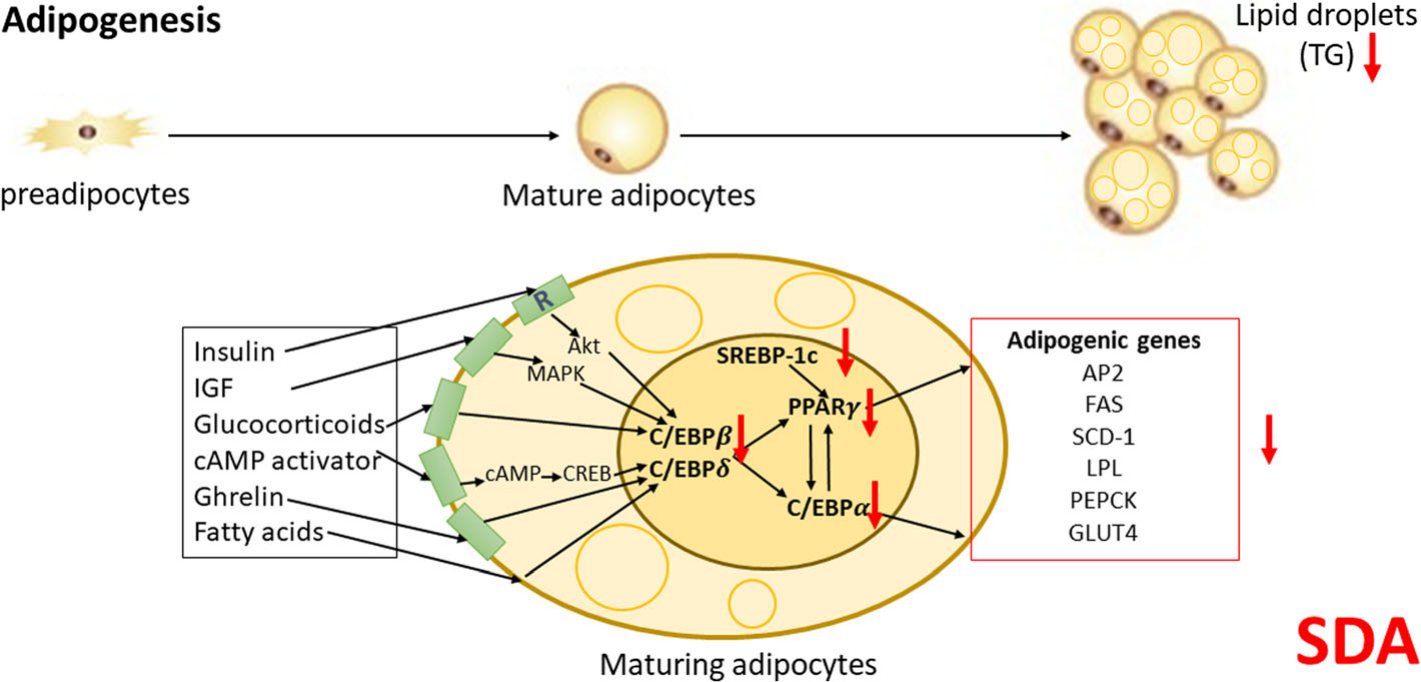
A summary of the effects of SDA on the transcriptional regulation of 3T3-L1 adipogenesis. SDA was able to inhibit adipocyte differentiation and reduce TG accumulation in 3T3-L1 adipocytes through down-regulation of certain adipogenic transcription factors and lipid accumulation genes

This study has demonstrated that SDA was able to inhibit adipocyte differentiation and reduce lipid accumulation in 3T3-L1 adipocytes through down-regulation of adipogenic transcription factors and lipid accumulation genes (Fig. 7). These findings warrant further investigation that will develop SDA as a natural and effective agent for the prevention or treatment of obesity.

## Additional file

Additional file 1: Table S1. Oligonucleotide primer sequences used in real-time PCR for adipogenic transcriptional factors and lipid accumulation genes. (DOCX 15 kb)

## Abbreviations

ALA: Alpha-linolenic acid
AP2: Adipocyte fatty acid-binding protein
C/EBP: CCAAT-enhancer-binding protein
FAS: Fatty acid synthase
GLUT: Glucose transporter
LPL: Lipoprotein lipase
PEPCK: Phosphoenolpyruvate carboxykinase
PPARγ: Peroxisome proliferator-activated receptor gamma
SCD: Stearoyl-CoA desaturase
SDA: Stearidonic acid
SREBP: Sterol regulatory element-binding protein
