# Supplementary material for: Suppression of adipocyte differentiation and lipid accumulation by stearidonic acid (SDA) in 3T3-L1 cells
Source: Lipids Health Dis. 2017 Sep 25;16:181. doi: 10.1186/s12944-017-0574-7 (PMC5613458; doi:10.1186/s12944-017-0574-7)
Supplement: Additional file 1: Table S1. — Oligonucleotide primer sequences used in real-time PCR for adipogenic transcriptional factors and lipid accumulation genes. (DOCX 15 kb) [file 12944_2017_574_MOESM1_ESM.docx]

**Response to Reviewers**

**Reviewer #1**

In the present manuscript, authors showed that SDA suppresses adipocyte differentiation and lipid accumulation in 3T3-L1 cells. They also showed down-regulation of adipogenic transcription factors and various genes acted upon by SDA. The studies are well designed and carefully conducted. The manuscript is well written.

1. Please include a model representing mechanism/pathway /genes inhibited by SDA for easy understanding of conclusions.

**Answers:**

Great idea! Thanks for the good advice. The Fig. 7 (Effects of SDA on the transcriptional regulation of 3T3-L1 adipogenesis) with a detailed and overall summary of this paper was added to the paper.

2. Some of the recent references are missing.

**Answers:**

Thanks for the suggestion. We carefully revised and edited the overall reference once again to make sure all the references are correct and not missing.

**Reviewer #2**

In this manuscript Li et al. analyzed the effect of on stearidonic acid (SDA) adipocyte differentiation. They demonstrated that SDA decrease the differentiation of 3T3-L1 cells to adipocytes via the lower expression of transcription factors and the transcription of genes related with adipogenesis. They also concluded that SDA is an effective precursor of EPA, but not of DHA in the cell. The aim of the study is clear and well justified. The manuscript is easy to follow, methods are appropriated and conclusions follow the results. There are only some remarks that should be addressed in order to improve the manuscript.

1. The introduction section is interesting but is too long. Try to condense it keeping the main information.

**Answers:**

We condensed the introduction from more than 3 pages to 2.5 pages. Thanks for the suggestion and it looks much cleaner than before.

2. PPAR$\gamma$ binding assay is confusing; in fact, it is a quantitative ELISA method using the response element for PPAR$\gamma$ adsorbed on the plaque. It is not really intended to measure the binding capacity (i.e to determine Kd constants) of PPAR$\gamma$. Include more information about the method considering that is just a quantitate method for PPAR$\gamma$.

**Answers:**

Thanks for this valuable correction. This assay is a quantitative ELISA assay. It measures the specific binding of activated PPARγ to the consensus sequence. In our purpose, we tried to measure how much of activated transcription factor PPAR$\gamma$ in the nuclear extracts at the transcriptional level after SDA treatment. Therefore, we changed our expression from “PPAR$\gamma$ binding activity” to “PPAR$\gamma$ transcriptional activity” and explained this is just an ELISA-based quantitative assay of activated PPAR$\gamma$. The relative text was all revised accordingly.

3. Cell viability was determined at 72h of incubation with SDA, whereas transcription of genes associated with adipogenesis and cell fatty acids were determined were assessed after 6 days. Are cells equally viable 6 days after the differentiation stimulus?

**Answers:**

This is a good question. Typically, the cell toxicity assay wouldn’t go that long (72h). We did 72h experiment to make sure the cell didn’t die after long time treatment. Besides, this cell line is special, after 72h, cells get into differentiation. After differentiation, the cells were always 100% confluence if no accident. As you can see in our Oil Red O staining on Day 6 (Fig. 3), the cells were 100% confluence. This is a proof by the side for the non-toxicity of SDA on 3T3-L1 cells.

4. As recognized by the Authors, results are quite controversial with a previous report (Reference 40); Authors argue that in their study the FA were added after the differentiation stimulus. However there are more differences between both studies that should be discussed (including FA doses) in order to expose a more general panorama about the effect of omega-3 FA on adipocyte differentiation.

**Answers:**

Thanks for the suggestion. We modified and discussed more about this question in the same paragraph (the second paragraph of Discussion). Actually, we did both 50 and 200 uM omega-3 FA treatment, while they did 50 uM treatment. In addition, in their study it seems that preadipocytes were not completely stimulated to mature adipocytes. So we add these in the text—

“One study published opposite results, in which they found EPA and DHA promoted the differentiation of 3T3-L1 preadipocytes as evidenced by increased lipid content [36]. However, they treated the 3T3-L1 cells not only during differentiation, but also 24-hour pretreatment before differentiation, so their results included both effects of EPA and DHA on differentiation and proliferation [36]. In our study, omega-3 fatty acids had an inhibitory effect on 3T3-L1 cell differentiation. It is not clear as to the effect of these omega-3 fatty acids on 3T3-L1 cell proliferation. In addition, our cells were much more differentiated than theirs. As shown in Fig. 3, there is a great significance between Non-diff control group and Diff control group. However, in their study, only a few 3T3-L1 preadipocytes were differentiated into mature adipocytes filled with lipid droplets based on their Bodipy staining and fluorescent images [36].”
